# Case Report: The role of nodal staging in periocular merkel cell carcinoma — lessons from three cases

**DOI:** 10.3389/fonc.2026.1935668

**Published:** 2026-08-24

**Authors:** Wojciech Adamski, Anna Rzeszotarska, Barbara Paprzycka

**Affiliations:** 1 Department of Ophthalmology, Regional Hospital in Poznan, The Greater Poland Specialist Center, Poznan, Poland; 2 Laboratory of Vision Science and Optometry, Faculty of Physics and Astronomy, Adam Mickiewicz University, Poznan, Poland

**Keywords:** adjuvant radiotherapy, cutler-beard flap, eyelid neoplasm, merkel cell carcinoma, neuroendocrine carcinoma, oculoplastic surgery, periocular tumour, sentinel lymph node biopsy

## Abstract

Merkel cell carcinoma (MCC) of the eyelid is a rare, aggressive cutaneous neuroendocrine malignancy. We report three patients with upper eyelid MCC managed at a single ophthalmology centre. Patients were aged 74, 78 and 65 years; two were female and one male. All underwent wide local excision and complex eyelid reconstruction using Cutler-Beard and adjunctive flap techniques. Outcomes differed substantially: the patient who underwent neither nodal staging nor adjuvant therapy developed parotid metastases within two months and died at 4.5 months. The two patients in whom nodal staging was performed remain disease-free at 35 and 13 months, respectively, despite also not receiving adjuvant radiotherapy. These cases illustrate the diagnostic challenge of periocular MCC and are consistent with guideline recommendations for nodal staging, although a series of three patients cannot separate the effect of staging from that of performance status.

## 1 Introduction

Merkel cell carcinoma (MCC) is a rare, aggressive cutaneous neuroendocrine malignancy that predominantly affects elderly, fair-skinned individuals. The head and neck region is the most common site, with the eyelid involved in approximately 2.5% of cases (1, 2). The tumour presents as a painless, rapidly expanding nodule; its resemblance to benign adnexal lesions such as chalazia or sebaceous cysts frequently leads to diagnostic delay.

Pathogenesis involves Merkel cell polyomavirus (MCPyV) integration in approximately 80% of cases (3). The AEIOU criteria (Asymptomatic, Expanding rapidly, Immune suppression, Older than 50, UV-exposed site) facilitate clinical recognition, with 89% of confirmed MCCs satisfying three or more features (4). Histopathological diagnosis requires characteristic perinuclear dot-like CK20 staining with TTF-1 negativity (1, 5). Although overall disease-specific mortality is 25–35%, eyelid MCC carries an approximately 10% disease-specific mortality rate, likely reflecting earlier specialist presentation (2).

Eyelid MCC has been reported mainly in pooled registry analyses and in cohorts assembled from dermatology or head and neck services, in which the periocular subgroup is small and the reconstructive detail limited (2, 5). What the present report adds is a consecutive, ophthalmology-managed series in which nodal staging differed between patients while the surgical approach and the decision to withhold adjuvant radiotherapy did not. The relation between staging and locoregional course can therefore be observed with the other principal variables held approximately constant. Reconstruction is reported in operative detail, including a combined Cutler-Beard, glabellar and modified Tenzel repair supplemented by hard palate mucosal grafting, which has not been described in the periocular literature. To the best of our knowledge this is the largest published series of eyelid MCC managed by an ophthalmology department in Poland, and one of few from Central and Eastern Europe dedicated to the periocular localisation of this tumour. We report three consecutive patients to illustrate how divergent management decisions translate into markedly different outcomes.

## 2 Case description

### 2.1 Case 1

**Figure 1:**
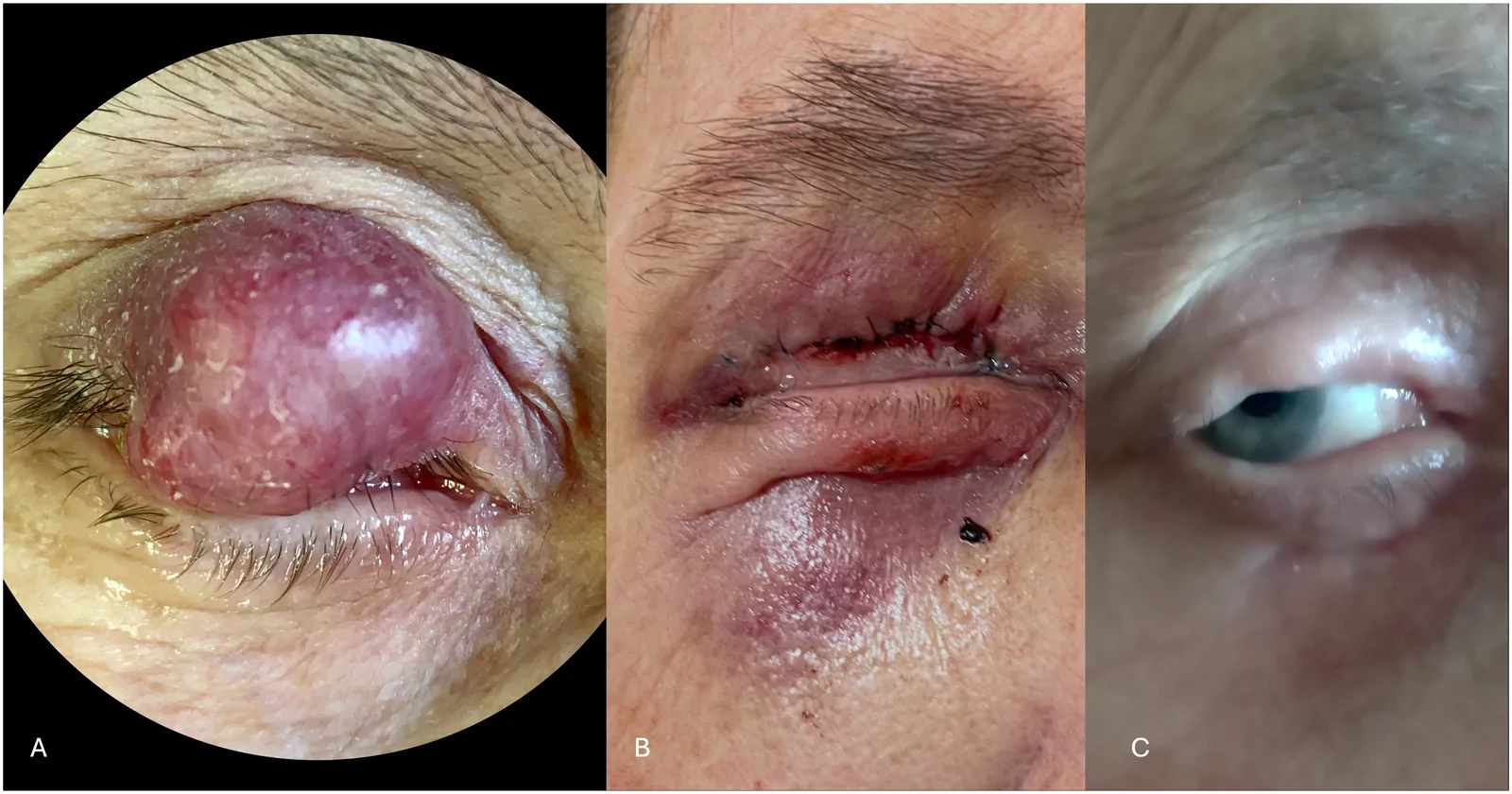
Case 1. **(A)** Preoperative appearance of the right upper eyelid lesion. **(B)** Intermediate appearance during the staged Cutler-Beard flap repair, prior to eyelid opening. **(C)** Final postoperative result.

A 74-year-old female presented with a fast-growing lesion on the right upper eyelid measuring 2.2 cm, present for eight weeks. The referring diagnosis was an eyelid tumour of undetermined type. She was not immunosuppressed. Staging imaging (head MRI, chest CT, abdominal and cervical ultrasound) revealed no metastatic disease. Wide local excision with a planned 10 mm margin was performed, followed by Cutler-Beard flap reconstruction. Immunohistochemistry was positive for synaptophysin, chromogranin A, pan-CK, EpCAM and CK20, and negative for TTF-1; the Ki-67 index was 60% (1, 5). Resection margins were histologically clear, with a minimum clearance of 8 mm, and neither lymphovascular nor perineural invasion was identified. Excisional regional lymph node biopsy showed no malignancy. Adjuvant therapy was withheld owing to overall health status. At 35 months, the patient remains disease-free (**Figure 1**).

### 2.2 Case 2

**Figure 2:**
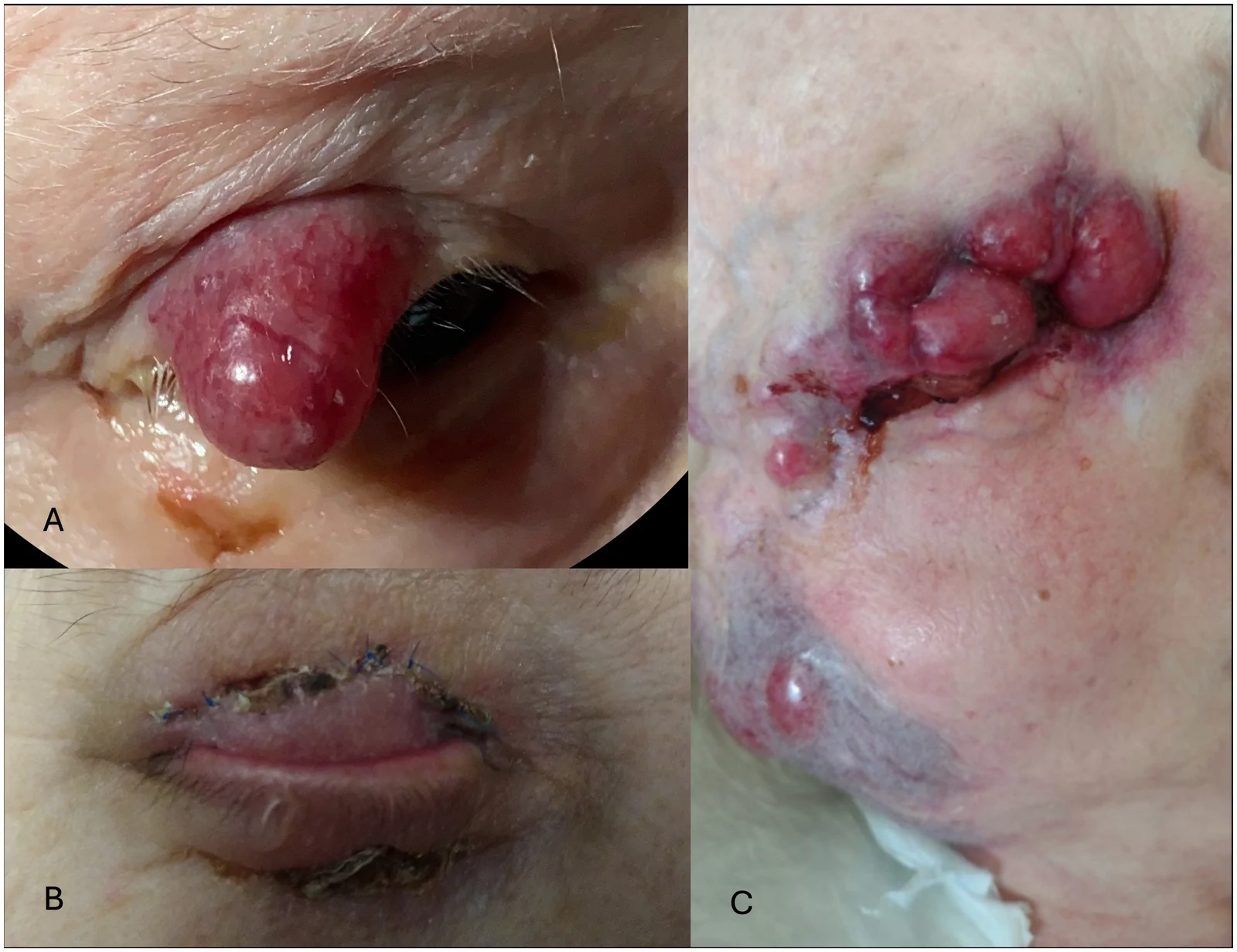
Case 2. **(A)** Clinical appearance at initial presentation. **(B)** Postoperative result before eyelid separation. **(C)** Appearance following the development of parotid nodal metastases.

A 78-year-old female presented with a rapidly enlarging right upper eyelid mass measuring 1.8 cm, present for eight weeks and treated elsewhere as a chalazion. She was not immunosuppressed. Staging imaging was negative for metastasis. Immunohistochemistry was positive for chromogranin A, pan-CK, EpCAM and CK20, with weak synaptophysin expression and TTF-1 negativity; the Ki-67 index was 65% (1, 5). Wide local excision with a planned 10 mm margin and modified Cutler-Beard flap reconstruction were performed. Resection margins were histologically clear, with a minimum clearance of 10 mm, and neither lymphovascular nor perineural invasion was identified. Nodal staging was not undertaken owing to compromised general health, which also precluded adjuvant therapy. Parotid lymph node metastases developed approximately two months postoperatively. The patient died of disease at 4.5 months (**Figure 2**).

### 2.3 Case 3

**Figure 3:**
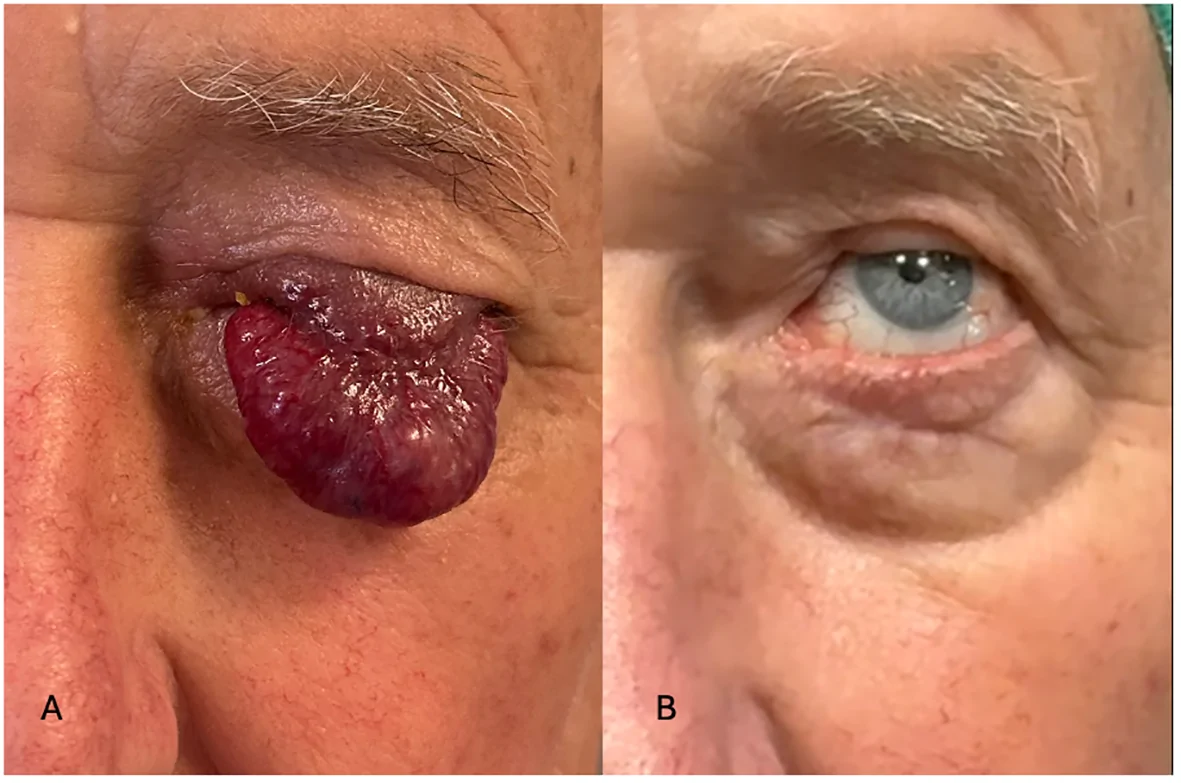
Case 3. **(A)** Preoperative appearance of the left upper eyelid mass. **(B)** Postoperative result following complex reconstruction with Cutler-Beard, glabellar and modified Tenzel flaps supplemented by a hard palate mucosal graft.

A 65-year-old male was admitted with a rapidly growing left upper eyelid mass measuring 2.4 cm, present for five weeks and initially diagnosed as a haemangioma. He was not immunosuppressed. Staging imaging revealed no metastasis. Wide local excision with a planned 10 mm margin was performed; reconstruction employed Cutler-Beard, glabellar and modified Tenzel flaps supplemented by a hard palate mucosal graft for tarsal reconstruction. Immunohistochemistry was positive for CK20 and chromogranin A and weakly for synaptophysin; the Ki-67 index was 50%, and TTF-1, S-100 and CD45 were negative (1, 5). Resection margins were histologically clear, with a minimum clearance of 10 mm, and neither lymphovascular nor perineural invasion was identified. Sentinel lymph node biopsy (SLNB) was negative (6). The patient declined adjuvant radiotherapy. At 13 months, he remains disease-free (**Figure 3**).

## 3 Clinical timeline

**Table 1** summarises patient characteristics, histopathology, management and outcome.

**Table 1:** Patient characteristics, histopathology, management and outcome.

Parameter	Case 1	Case 2	Case 3
Age (years), sex	74, female	78, female	65, male
Site	Right upper eyelid	Right upper eyelid	Left upper eyelid
Duration before presentation	8 weeks	8 weeks	5 weeks
Initial clinical diagnosis	Eyelid tumour, type undetermined	Chalazion	Haemangioma
Immunosuppression	Absent	Absent	Absent
Maximum tumour diameter	2.2 cm	1.8 cm	2.4 cm
Staging imaging	Negative	Negative	Negative
AJCC 8th edition stage*	pT2 pN0 cM0, stage IIA	cT1 cN0 cM0, clinical stage I	pT2 pN0(sn) cM0, stage IIA
Planned excision margin	10 mm	10 mm	10 mm
Histological margin clearance	Clear, 8 mm	Clear, 10 mm	Clear, 10 mm
Lymphovascular invasion	Not identified	Not identified	Not identified
Perineural invasion	Not identified	Not identified	Not identified
CK20	Positive, dot-like	Positive, dot-like	Positive, dot-like
TTF-1	Negative	Negative	Negative
Additional immunohistochemistry	Synaptophysin+, chromogranin A+, pan-CK+, EpCAM+	Chromogranin A+, pan-CK+, EpCAM+, synaptophysin weak	Chromogranin A+, synaptophysin weak, S-100−, CD45−
Ki-67 index	60%	65%	50%
Reconstruction	Cutler-Beard flap	Modified Cutler-Beard flap	Cutler-Beard, glabellar and modified Tenzel flaps with hard palate mucosal graft
Nodal staging	Excisional regional lymph node biopsy, negative	None	Sentinel lymph node biopsy, negative
Adjuvant radiotherapy	Not given (unfit)	Not given (unfit)	Declined by patient
Recurrence	None	Parotid nodal metastases at 2 months	None
Follow-up	35 months	4.5 months	13 months
Outcome	Alive, disease-free	Died of disease	Alive, disease-free

*Assigned retrospectively according to the 8th edition of the AJCC staging system for Merkel cell carcinoma. Case 2 was staged clinically, since no pathological nodal evaluation was performed; the nodal status of Case 1 rests on excisional regional lymph node biopsy rather than on sentinel lymph node biopsy.

CK, cytokeratin; EpCAM, epithelial cell adhesion molecule; sn, sentinel node; TTF-1, thyroid transcription factor 1.

## 4 Diagnostic assessment and therapeutic intervention

In all three patients, the clinical presentation prompted wide local excision without prior incisional biopsy. MCC was diagnosed on definitive histopathological examination of the excised specimen, with immunohistochemistry demonstrating characteristic perinuclear dot-like CK20 positivity and TTF-1 negativity in each case (1, 5). Staging work-up comprising MRI of the head, CT of the chest and abdomen, and cervical ultrasound was completed in all three cases and was negative for distant metastasis at presentation. Excision with a planned margin of 10 mm was performed in all patients, and margins were reported histologically clear in every case, with a minimum clearance of 8 mm in Case 1 and 10 mm in Cases 2 and 3. Reconstruction was individualised to the extent of the eyelid defect: a Cutler-Beard flap was used in Cases 1 and 2, and a combined Cutler-Beard, glabellar and modified Tenzel flap with hard palate mucosal graft was used in Case 3. Nodal staging by SLNB was performed in Case 3; regional lymph node biopsy was performed in Case 1; no nodal staging was performed in Case 2. None of the patients received adjuvant radiotherapy or systemic therapy.

## 5 Discussion

### 5.1 Differential diagnosis and diagnostic pitfalls

The clinical appearance of eyelid MCC is non-specific, and the first impression is usually benign. Chalazion, epidermal inclusion cyst, pyogenic granuloma and amelanotic naevus account for most initial misdiagnoses, and the resulting median delay from presentation to biopsy is approximately three months (1, 2). The referring diagnoses in the present series were chalazion, haemangioma and an eyelid tumour of undetermined type, and symptom duration before presentation was eight, eight and five weeks. All three lesions were excised on clinical suspicion of malignancy without prior incisional biopsy, and MCC was diagnosed only on the definitive specimen. Each patient satisfied four of the five AEIOU features, immune suppression being absent in all three, which exceeds the threshold of three features met by 89% of confirmed cases (4).

The malignant differential is the more consequential one. Basal cell carcinoma accounted for 158 of 174 malignant eyelid tumours (90.8%) in a population-based incidence cohort, and squamous cell carcinoma for a further 15 (8.6%) (7). A rapidly enlarging eyelid nodule is therefore far more likely to represent one of these tumours than MCC. Sebaceous carcinoma shares the elderly age profile and the upper eyelid predilection of MCC and may also present as a painless nodule; deep biopsy is often required, and special histological stains support the distinction (8). Sweat gland carcinomas, ocular adnexal lymphoma and eyelid deposits from a visceral primary complete the group of lesions that cannot be separated from MCC on clinical grounds alone (1, 5).

Rare adnexal malignancies of the periocular and facial skin present the same problem from the pathological side. Squamoid eccrine ductal carcinoma is one example. It overlaps histologically with squamous cell carcinoma, and shows biphasic differentiation, deep infiltration and a high rate of perineural invasion. No standardised treatment protocol exists, so misclassification at first assessment is common and definitive management is often delayed (9). The parallels with MCC are close. Both tumours are rare enough that the discriminating immunohistochemical panel is requested only when the diagnosis is already suspected, and both are managed by wide excision with consideration of Mohs micrographic surgery and adjuvant radiotherapy (9). Awareness of this wider group of aggressive adnexal tumours may shorten the interval to definitive histopathology.

Immunohistochemistry resolves most of this differential. Perinuclear dot-like CK20 positivity with TTF-1 negativity distinguishes MCC from metastatic small cell lung carcinoma, in which TTF-1 is usually positive (1, 5). CD45 negativity excludes lymphoma and S-100 negativity excludes amelanotic melanoma; both were documented in Case 3. The proliferation index was high in all three tumours, at 60%, 65% and 50% in Cases 1 to 3 respectively. Ki-67 is characteristically elevated in MCC and supports the diagnosis, but no validated prognostic threshold has been established, and the two patients with the highest indices had opposite outcomes. Because none of these panels is requested unless a malignant diagnosis is entertained, the decision to biopsy remains the limiting step in the diagnostic pathway.

### 5.2 Nodal staging

Comprehensive nodal staging is critical. Whole-body FDG-PET/CT or contrast-enhanced CT detects occult metastases in 12–20% of clinically node-negative patients (10, 11). SLNB is recommended for all patients without clinically evident nodal disease. In head and neck MCC, sensitivity is approximately 52.6% owing to aberrant lymphatic drainage; 26.7% of patients with a negative SLNB subsequently develop regional recurrence (12).

The distinction between clinical and pathological nodal status is central to the courses observed here. Cases 1 and 3 were pathologically node-negative and were assigned stage IIA, whereas Case 2 was staged clinically as stage I on the basis of negative imaging alone. Case 2 underwent no nodal staging and developed parotid nodal metastases within two months, with death at 4.5 months. The interval suggests occult nodal disease present at operation, although interval progression cannot be excluded. This sequence is consistent with the rationale for routine SLNB set out in current guidance (6, 10, 11). It cannot, however, be attributed to the omission of staging alone. The same patient was considered unfit for both nodal staging and adjuvant therapy, and reduced performance status is itself associated with adverse outcome in MCC. The observation is therefore compatible with confounding by indication, and should be read as illustrative rather than as evidence of causation.

### 5.3 Surgical management

Wide local excision with 1–2 cm margins is the surgical standard. In the periocular setting, narrower margins are acceptable when adjuvant radiotherapy is planned (11, 13). Mohs micrographic surgery (MMS) represents an established alternative, offering complete circumferential margin assessment while preserving periocular tissue. Globe preservation has been reported in up to 78% of periocular MCC cases treated with MMS (5, 14). Population-based data suggest that overall survival after MMS is comparable to or exceeds that after wide local excision for early-stage disease (15). Although MMS was not used in this series, it should be considered in suitable patients.

Local recurrence after histologically complete excision may be less frequent than historically assumed. Local recurrence occurred in 8 of 447 patients with clinically localised MCC excised with pathologically negative margins, giving a 1-year rate of 1.8% (95% CI 0.8–3.6%); all events arose among the 393 patients treated by surgery alone (16). The authors of that series concluded that their data do not support routine adjuvant radiotherapy after complete excision of a primary tumour (16). Cases 1 and 3 were excised with clear margins, received no adjuvant radiotherapy, and remain free of local recurrence at 35 and 13 months respectively, which is consistent with those figures.

### 5.4 Adjuvant radiotherapy

Adjuvant radiotherapy is recommended for tumours greater than 1 cm, lymphovascular invasion, close or positive margins, a positive SLNB result, immunosuppression, or head and neck location (10, 11). Meta-analysis demonstrates improvement in overall survival (hazard ratio 0.81) and disease-free survival (hazard ratio 0.45) with adjuvant radiotherapy, reducing local recurrence by approximately 80% and regional recurrence by approximately 70% (17). Early initiation within 7–8 weeks of surgery is associated with improved outcomes (11, 13).

None of the patients in this series received adjuvant radiotherapy. Omission of adjuvant therapy has been associated with accelerated locoregional progression in registry and cohort data (18–20), whereas recent negative-margin data place the local recurrence risk after surgery alone below 2% (16). The indication in periocular MCC therefore remains a matter of individual assessment, weighing tumour size, margin status, nodal findings and ocular surface toxicity against a modest absolute gain in local control. In Cases 1 and 3 the margins were clear, the nodes pathologically negative, and lymphovascular and perineural invasion absent; tumour size above 1 cm and head and neck location were the only remaining indications for adjuvant treatment. For resectable advanced disease, the most recent NCCN guidelines also recognise neoadjuvant immunotherapy as an emerging option, with trials demonstrating pathological response rates that may reduce surgical morbidity (11).

One of the three patients in this series died of disease. Pooled eyelid MCC cohorts report a disease-specific mortality of approximately 10% (2), and a recent periocular cohort reported 2-year survival of 88.9% where multidisciplinary management was applied consistently (14). The single death recorded here occurred in the patient who was unfit for both nodal staging and adjuvant therapy, which is consistent with published data associating incomplete staging and omission of adjuvant therapy with adverse outcomes.

### 5.5 Implications for clinical practice

Several practical implications follow from this series and from the guidelines against which it is measured. An eyelid nodule that enlarges over weeks in a patient older than 50 years warrants excision or biopsy, with a neuroendocrine immunohistochemical panel requested at the outset. The AEIOU features provide a workable trigger for that decision (4).

Nodal staging should be planned at the time the diagnosis is made rather than after reconstruction. A Cutler-Beard flap and the adjunctive flaps used in Case 3 alter periocular lymphatic drainage, and delayed sentinel node mapping in a reconstructed eyelid may be unreliable (6). Unfitness for adjuvant radiotherapy is not in itself a reason to omit nodal staging; SLNB is a short procedure, and a positive result alters surveillance intensity even where treatment options are otherwise limited (6, 11).

Referral to a multidisciplinary board covering ophthalmic oncology, dermatology, head and neck surgery, radiation oncology and pathology should be routine rather than reserved for advanced disease (11, 14). Where adjuvant radiotherapy is declined, as in Case 3, the reason should be documented and the recommendation revisited at each follow-up visit. Because randomised evidence confined to periocular MCC is unlikely to become available, prospective multicentre registry data and translational work on MCPyV status and immune correlates offer the most realistic route to firmer recommendations (3, 11).

### 5.6 Limitations

Three patients cannot support statistical analysis, and the findings should be read as descriptive.

Selection bias operates at several levels. All three patients were referred to a tertiary ophthalmology unit with a lesion already considered surgical, so patients managed elsewhere, treated non-surgically, or lost before referral are not represented. Case inclusion was retrospective and depended on the histopathological diagnosis being coded correctly, which may under-represent tumours initially classified as squamous cell carcinoma.

Treatment was heterogeneous by circumstance rather than by protocol. Nodal staging was performed by excisional regional lymph node biopsy in Case 1, by SLNB in Case 3, and not at all in Case 2. These are not equivalent procedures; excisional biopsy of a clinically selected node does not reliably sample the true drainage basin, and the two staged patients are therefore not directly comparable to each other. Adjuvant radiotherapy was withheld in every patient, for unfitness in two and by patient preference in one, which removes the comparison that would otherwise be most informative.

Confounding by indication is the principal limitation of the central observation. The decision to omit nodal staging in Case 2 was itself driven by poor performance status, and poor performance status is independently associated with adverse outcome in MCC. The difference in course between Case 2 and the other two patients cannot be attributed to nodal staging alone. Follow-up also differed substantially, at 35, 13 and 4.5 months; the two surviving patients have not been observed across the interval in which most MCC recurrences arise, and the disease-free status reported here may not persist.

Staging was assigned retrospectively, and neither the nodal nor the tumour category is equally secure across patients. Case 2 was staged clinically, and the pN0 of Case 1 rests on excisional biopsy of a regional node rather than on sentinel node mapping. All three tumours were removed by full-thickness eyelid resection, so orbicularis muscle was contained within every specimen; invasion of muscle was not described in any histopathological report, and the T category was assigned on tumour diameter accordingly. Generalisability is further limited by the single-centre design, by the small number of patients, and by the absence of MCPyV testing, which is not routinely available at the treating institution.

## 6 Conclusions

A high index of clinical suspicion is required for the early diagnosis of eyelid MCC, since the tumour resembles both benign adnexal lesions and the commoner eyelid malignancies. The observations reported here complement, rather than independently validate, an existing evidence base. Current guidance supports wide local excision, nodal staging by sentinel lymph node biopsy, and selective use of adjuvant radiotherapy (6, 10, 11, 17). A series of three patients cannot establish a causal relation between omission of nodal staging and adverse outcome; the divergent courses observed here are equally compatible with differences in performance status at presentation. Where management follows multidisciplinary guidelines, the available evidence suggests that eyelid MCC carries a favourable prognosis, with functional globe preservation achievable in most patients (2, 14).

## 7 Patient perspective

Case 1: The patient reported initial reassurance after a benign diagnosis was first suggested by a general practitioner. Delay of several weeks occurred before ophthalmological referral. She expressed satisfaction with the functional outcome of eyelid reconstruction and remains under regular oncological surveillance.

Case 3: The patient was informed of the recommendation for adjuvant radiotherapy following surgery and negative sentinel node biopsy, but declined owing to personal preference. He remains under close follow-up and has been re-counselled regarding the option of radiotherapy should any sign of recurrence emerge.

## Data Availability

The original contributions presented in the study are included in the article/**Supplementary Material**. Further inquiries can be directed to the corresponding author.

## Supplementary material

Supplementary File CARE checklist.
