# Supplementary material for: Case Report: The role of nodal staging in periocular merkel cell carcinoma — lessons from three cases
Source: Front Oncol. 2026 Aug 24;16:1935668. doi: 10.3389/fonc.2026.1935668 (PMC13547002; doi:10.3389/fonc.2026.1935668)
Supplement: Supplementary file 1 [file Supplementaryfile1.pdf]

CARE Checklist

Manuscript: "Case Report: The role of nodal staging in periocular merkel cell carcinoma — lessons from three cases" (Adamski W, Rzeszotarska A, Paprzycka B). Frontiers in Oncology,

Topic	Item	Checklist item description	Reported in section
Title	1	The diagnosis or intervention of primary focus followed by the words "case report".	Title. "Case Report: The role of nodal staging in periocular merkel cell carcinoma — lessons from three cases"
Key Words	2	2 to 5 key words that identify diagnoses or interventions in this case report, including "case report".	Keywords section. Eight keywords identifying the diagnosis and the interventions. The article type is declared as Case Report and the words appear in the title.
Abstract	3a	Introduction: What is unique about this case? What does it add to the medical literature?	Abstract, opening sentences.
	3b	The patient's main concerns and important clinical findings.	Abstract.
	3c	The main diagnoses, interventions, and outcomes.	Abstract.
	3d	Conclusion — What are the main "take-away" lessons from this case?	Abstract, closing sentences.
Introduction	4	Brief background summary of this case referencing the relevant medical literature.	Section 1 (Introduction).
Patient Information	5a	De-identified patient specific information.	Sections 2.1, 2.2 and 2.3; Table 1 (age, sex, site). No identifying details are reported.
	5b	Primary concerns and symptoms of the patient.	Sections 2.1–2.3; Table 1 (duration before presentation, maximum tumour diameter).
	5c	Medical, family, and psycho-social history including relevant genetic information.	Sections 2.1–2.3; Table 1. Immunosuppression status is reported for each patient. Family and psycho-social history were not relevant to the presentation and are not reported.
	5d	Relevant past interventions with outcomes.	Sections 2.2 and 2.3 (prior management elsewhere as chalazion and as haemangioma respectively); Table 1 (initial clinical diagnosis).
Clinical Findings	6	Describe significant physical examination (PE) and important clinical findings.	Sections 2.1–2.3; Table 1; Figures 1A, 2A and 3A.
Timeline	7	Historical and current information from this episode of care organized as a timeline.	Section 3 (Clinical timeline) with Table 1.
Diagnostic Assessment	8a	Diagnostic testing (such as PE, laboratory testing, imaging, surveys).	Section 4; Table 1 (staging imaging, immunohistochemistry, Ki-67, margin status).

	8b	Diagnostic challenges (such as access, financial, or cultural).	Section 5.1 (Differential diagnosis and diagnostic pitfalls).
	8c	Diagnostic reasoning including other diagnoses considered.	Section 5.1; Section 4.
	8d	Prognostic characteristics (such as staging) where applicable.	Table 1 (AJCC 8th edition stage, Ki-67, nodal status); Sections 5.2 and 5.6.
Therapeutic Intervention	9a	Types of intervention (such as pharmacologic, surgical, preventive, self-care).	Sections 2.1–2.3 and Section 4; Table 1 (excision, reconstruction, nodal staging).
	9b	Administration of intervention (such as dosage, strength, duration).	Sections 2.1–2.3 and Section 4 (planned and achieved excision margins, reconstructive technique, nodal staging procedure).
	9c	Changes in intervention (with rationale).	Sections 2.2 and 2.3; Section 4 (adjuvant radiotherapy withheld for unfitness in two patients and declined by the patient in one).
Follow-up and Outcomes	10a	Clinician and patient assessed outcomes.	Sections 2.1–2.3; Table 1; Section 7 (Patient perspective); Figures 1C, 2C and 3B.
	10b	Important follow-up diagnostic and other test results.	Sections 2.1–2.3; Table 1 (recurrence, follow-up duration, outcome).
	10c	Intervention adherence and tolerability (How was this assessed?).	Sections 2.3 and 7 (declined adjuvant radiotherapy, documented at follow-up and revisited at each visit).
	10d	Adverse and unanticipated events.	Section 2.2 (parotid nodal metastases at two months, death of disease at 4.5 months); Figure 2C.
Discussion	11a	A scientific discussion of the strengths AND limitations associated with this case report.	Section 5, with limitations set out in Section 5.6.
	11b	Discussion of the relevant medical literature with references.	Sections 5.1–5.5.
	11c	The scientific rationale for any conclusions (including assessment of possible causes).	Sections 5.2, 5.4 and 5.6; Section 6 (Conclusions).
	11d	The primary “take-away” lessons of this case report.	Section 5.5 (Implications for clinical practice); Section 6.
Patient Perspective	12	The patient should share their perspective in one to two paragraphs on the treatment(s) they received.	Section 7 (Patient perspective), for Cases 1 and 3.
Informed Consent	13	Did the patient give informed consent? Please provide if requested.	Yes. Ethics statement.

The CARE guidelines are available at https://www.care-statement.org.
